# Community Health Empowerment Through Clinical Pharmacy: A Single-Arm, Post-Intervention-Only Pilot Implementation Evaluation

**DOI:** 10.3390/pharmacy13050141

**Published:** 2025-10-01

**Authors:** Clipper F. Young, Casey Shubrook, Cherry Myung, Andrea Rigby, Shirley M. T. Wong

**Affiliations:** 1 Department of Clinical Sciences and Community Health, College of Osteopathic Medicine, Touro University California, 1310 Club Drive, Vallejo, CA 94592, USA; 2 College of Osteopathic Medicine, Touro University California, 1310 Club Drive, Vallejo, CA 94592, USA; cshubroo@student.touro.edu (C.S.);; 3 College of Pharmacy, Touro University California, Vallejo, CA 94592, USA

**Keywords:** medication therapy management, seniors, self-advocacy, chronic disease management, community health

## Abstract

Background: The Pharm2Home Initiative’s Community Health Arm adopts a health-equitable approach to chronic disease education and medication therapy management (MTM). We serve senior residents of Solano County, California, who live in affordable housing and have limited financial resources. Aim: This evaluation assesses the uptake of chronic disease management recommendations provided by clinical pharmacists during MTM sessions at community events. Methods: The program engaged clinical pharmacists to provide tailored education and healthcare interventions in senior housing facilities. The goal was to empower seniors to manage their health effectively. The sessions covered various topics, including expired or duplicated medications, incorrect medication use, consultations on medication management, immunizations, and lifestyle adjustments. Results: Over an 18-month period, from January 2022 to August 2023, the program involved 65 participants across ten community health events. These events provided approximately 65 h of direct intervention. Many participants reported significant improvements in understanding their treatment plans and navigating their health needs more confidently. Feedback from 60 seniors after the sessions indicated that 88% felt much better informed about their medications, and 75% expressed that their concerns were addressed extremely well. Conclusions: These outcomes demonstrate the importance of clinical pharmacist-led interventions in improving seniors’ medication use and chronic disease management. The initiative’s approach advocates for integrating clinical pharmacists into community health settings, suggesting a scalable model for enhancing person-centered care. However, further studies are necessary to assess the long-term impacts of these interventions and explore their effectiveness across diverse age groups and more complex conditions.

## 1. Introduction

According to the National Council on Aging (NCA), 56 million Americans are 65 years or older, with nearly 95% experiencing at least one chronic condition, such as diabetes, arthritis, or heart disease [1]. Additionally, 80% of this population have two or more chronic conditions. Furthermore, the National Assessment of Adult Literacy (NAAL), conducted by the Centers for Disease Control and Prevention (CDC), revealed that 71% of adults aged 60 and above faced challenges in utilizing printed materials; 80% encountered difficulties with documents featuring forms and charts; and 68% experienced challenges in interpreting numbers and performing calculations [2]. For these reasons, a grassroots intervention is warranted to enhance older adults’ health literacy and their approach to self-advocacy for promoting healthy aging [3].

Pharmacists’ expanding role in the healthcare team has positioned us as accessible resources, particularly in underserved areas, creating the necessity for the role to grow [4]. Due to their unique position, pharmacists can build a bridge between clinical care and public health. For instance, in 2008, pharmacists in Georgia developed an outreach program to improve medication and disease state knowledge in the community, as patient education was identified as a critical factor in enhancing not only medication adherence but also increasing comfort when community members discussed their health with healthcare providers [5]. In 2007, the primary care mental health integration service was established nationwide in Veterans Affairs medical centers, which addressed post-traumatic stress disorder (PTSD), mild to moderate depression, and anxiety [6]. Clinical pharmacists utilized telephone assessments, which reduced the travel costs for their patients and improved mental health outcomes by the end of the 12-week follow-up period [6]. Those with mild to moderate mental health conditions showed improvement, and interdisciplinary collaboration was promoted [6].

Beyond the context of the United States, chronic conditions are the primary cause of morbidity and mortality globally. Noncommunicable diseases (NCDs) account for approximately 74% of all global fatalities, and 86% of premature deaths (defined as those occurring before the age of 70) are prevalent in low- and middle-income countries [7]. This disparity highlights substantial inequities in prevention and management strategies. Across OECD countries, more than one-third of the population reports a chronic illness. Diabetes affects approximately 6.9% of adults on average, with prevalence exceeding 10% in Mexico, Turkey, Chile, the United States, and Spain [8]. This highlights substantial cross-national burden and variation. Multimorbidity exhibits a pronounced increase with age. A comprehensive systematic review conducted in 2023, encompassing data from 54 countries, revealed that over 50% of adults aged above 60 are afflicted with two or more chronic conditions; the global pooled prevalence of multimorbidity was estimated to be 51% (95% CI, 44–58%) [9]. Regional estimates varied, with Asia reporting approximately 35% and South America experiencing a prevalence of around 46% [9]. Globally, limited health literacy is a prevalent issue. The eight-country European Health Literacy Survey revealed that 47% of adults exhibited “limited” health literacy, characterized by insufficient or problematic knowledge and skills [10]. Furthermore, systematic reviews consistently demonstrate an association between advanced age and diminished health literacy [11].

Medication Therapy Management (MTM) services have emerged as a crucial element in this ongoing evolution, enhancing patient care outcomes. These billable services improve the effectiveness and safety of medication regimens while also reducing healthcare-related expenses, such as unexpected medical visits and emergency department admissions. An example is our Community Health Arm (CHA) of the Pharm2Home (P2H) Initiative, which enables participants to deepen their understanding of their chronic health conditions and pharmacotherapies, including prescribed medications, over-the-counter medications, and supplements. By emphasizing health literacy, our program aims to enhance participants’ self-efficacy and self-advocacy in managing multiple chronic conditions, thereby addressing a care gap for this vulnerable population.

Our single-arm, post-intervention-only pilot implementation evaluation aimed to quantify the uptake of chronic disease management recommendations provided by clinical pharmacists during MTM sessions at community events. Thus, the focus was on feasibility and acceptability, rather than effectiveness testing. These recommendations include educating and recommending therapeutic modifications to optimize safety and effectiveness, addressing healthcare-related financial barriers, encouraging behavioral changes, and meeting patient-centered goals for senior residents in Solano County who live in affordable housing with limited income. We prioritized seniors residing in affordable housing due to their high-risk, underserved status, characterized by greater comorbidities, disabilities, and healthcare utilization. Additionally, the availability of housing sites facilitates efficient, place-based delivery of clinical pharmacy services.

## 2. Materials and Methods

### 2.1. Setting

The design of this evaluation was informed by the design of the intervention. The **Pharm2Home Initiative** is a clinical pharmacist-led program to enhance chronic disease management and pharmacotherapy utilization among medically underserved populations in Solano County, California. Pharm2Home seeks to reduce health disparities and promote health equity by extending care beyond clinic walls while generating actionable insights for broader dissemination and replication.

### 2.2. The Intervention

The CHA commenced operations in January 2022 in response to the fact that many individuals served by the Clinical Arm of the Initiative (initiated in January 2020) were not patients of our partner, the Solano County Family Health Services clinic system, and therefore did not benefit from our clinical pharmacy services.

The initial efforts of the CHA exposed seniors to enhanced medication management and a deeper understanding of their chronic conditions. The intervention was delivered through community health events, where clinical pharmacists provided one-on-one consultations, averaging 1 to 1.5 h per encounter. The overall goal of the encounters was to empower participants to raise their healthcare-related concerns with their healthcare providers during subsequent clinic visits, thereby enhancing their self-advocacy skills. Our consultation services aimed to (1) educate seniors about their chronic conditions and pharmacotherapies and (2) evaluate the safety and effectiveness of their current chronic disease pharmacotherapies. Participants left with a physical copy of our recommendations to share with their healthcare providers. Recommendations were made and supported by the most up-to-date clinical guidelines and evidence, utilizing an evidence-based, personalized approach.

The services provided were operationalized utilizing the Pharmacists’ Patient Care Process (PPCP)—Collect, Assess, Plan, Implement, a Follow-Up—as a practice framework, in conjunction with a universal precaution approach to health literacy (plain language instruction and teach-back for all participants) [12].

A standardized process was used to deliver the MTM consultation sessions to the participants. Here is the seven-step process:

1. Preparation: Thoroughly reviewed the medication lists (if provided) by the participant, including medication bottles and any associated records. If available, noted the participant’s renal and liver functions.
2. Medication Reconciliation (Collect): Compiled a comprehensive medication history, including prescriptions, over-the-counter medications, supplements, doses, frequencies, indications, adherence routines, and potential barriers to adherence. A history intake form and a clinical note template were developed to serve this purpose (See Supplemental Materials for more details).
3. Clinical Assessment (Assess):
  a. Identified drug-related issues:
    i. Indication: Determined the appropriate use of the drug based on the participant’s medical conditions.
    ii. Effectiveness: Evaluated the drug’s efficacy in treating the participant’s symptoms.
    iii. Safety: Assessed the potential risks and side effects of the drug.
    iv. Adherence: Asked about the participant’s adherence to the prescribed medication regimen.
  b. Applied explicit criteria for appropriateness:
    i. AGS Beers: Ensured that the participant was not taking prescribed medications on this list.
    ii. Renal dose adjustments: Monitored the participant’s renal/liver functions and recommended adjustments to medication dosage as necessary.
    iii. Major interactions: Identified potential interactions between the prescribed medication and other medications the participant was taking.
    iv. Duplication: Verified that the participant is not taking two medications within the same class or with a similar mechanism of action.
4. Education and Problem-Solving (Plan): Used plain language counseling, provided large-print handouts (if available), used pictograms, and facilitated teach-back to reinforce understanding. Addressed any capability or opportunity barriers (e.g., cost, accessibility, regimen complexity); offered pill organizers when feasible. The encounters were conducted in English, and for those who spoke a language other than English, a family member was present or a staff member from the site assisted with translation.
5. Action Plan Sharing (Implement): Collaborated on a concise action plan, involving shared decision-making, to outline agreed-upon action steps, monitoring mechanisms, warning indicators, and tasks for the upcoming PCP discussion.
6. Documentation: Completed the structured template at the end of the day, particularly focusing on a concise summary (medication regimen modification recommendations and rationale, Beers/interaction flags, monitoring requests) that would assist the participant in communicating with their PCP. This post-encounter clinical note was either emailed or mailed to the participant within one week after the encounter.
7. Follow-Up Call to Inquire about the Acceptance of Recommendations (Follow-Up): A standardized follow-up call would be attempted within four weeks (up to three attempts at different times) by a clinical pharmacist. This call would be utilized to assess the participants’ uptake of our recommendations, including the implementation and discussion of these recommendations during the PCP’s office visit.

### 2.3. Participants and Data Sources

#### 2.3.1. Inclusion and Exclusion Criteria

Inclusion criteria. Individuals were eligible if they:

1. Managed one or more chronic diseases (self-reported);
2. Resided in a low-income senior housing facility within Solano County, CA at the time of the event; and
3. Provided informed consent to participate in the MTM encounter and surveys administered at the community events.

Exclusion criteria. Individuals were excluded if they:

1. Were not residents of the partnering senior housing sites;
2. Declined or were unable to provide consent;
3. Were acutely unwell and required urgent medical attention at presentation.

#### 2.3.2. Data Collection

Encounters were conducted by a team of clinical pharmacists, pharmacy residents, and pharmacy and osteopathic medical students. Participants were introduced to the program, and consent forms were obtained, allowing the option to decline participation at any point during the encounter.

During each event, clinical pharmacists and trainees utilized a standardized, structured intake form to meticulously document the following information: (1) demographics; (2) chronic conditions; and (3) a comprehensive medication reconciliation, including all prescription, over-the-counter, and supplement items. Additionally, this intake form served as a standardization anchor point for the intervention process. This instrument is provided in the Supplemental Material as “Intake Form.” At the point of care, medication-related issues were categorized using a predefined taxonomy (e.g., expired medication, untreated condition, therapeutic duplication, medication without indication, category D drug–drug interaction, contraindication, Beers Criteria medication, and adverse drug reaction). The types of consultations delivered (e.g., medication, immunization, lifestyle) were recorded using the same form. This instrument was developed by the study team for this program and has not been previously validated. We acknowledge this limitation as a study weakness in the Bias section.

Baseline knowledge and understanding of their medications were assessed by asking each participant about their medications one at a time (e.g., what they are taking it for, how they are taking it, how often, and what they do if a dose is missed). After reviewing all the medications, the clinical pharmacist identified those that the participant seemed uncertain about, providing targeted education through handouts that included clarifications and recommendations. After gathering subjective and objective information, the participants’ health information was analyzed at the end of each encounter. The health information was assessed by applying each disease state’s clinical practice guidelines (e.g., the American Diabetes Association’s Standards of Care in Diabetes), leveraging the expertise of clinical pharmacists. After the encounter, closed-ended questions were immediately asked regarding how they felt about their knowledge and understanding of medications and medical conditions.

Often, the quality of the encounter—and the robustness of the data collected—directly depended on the information the participant was willing to share with the clinical pharmacy team (e.g., consisting of a clinical pharmacist and a student pharmacist) at that moment. The richness of the encounter and the recommendations were based on the amount of information the team had received and could potentially analyze. No incentives were given to any participants, and their participation was voluntary. The routinely collected data (January 2022 to August 2023), capturing one intervention encounter per participant, was initially used for program evaluation purposes, making our study a pilot implementation evaluation.

### 2.4. Medication Appropriateness

We evaluated potentially inappropriate medication (PIM) use by employing the 2023 American Geriatrics Society (AGS) Beers Criteria^®^, a comprehensive and consensus-driven list of medications that are generally recommended to be avoided by older adults, either broadly or in specific clinical circumstances [13]. The 2023 update organizes recommendations into five tables: medications to avoid, medications to avoid in specific diseases/syndromes, medications to use with caution, drug–drug interactions to avoid, and medications that require dose adjustment or avoidance with reduced kidney function [13].

We selected the AGS Beers Criteria because it is the most widely used tool for evaluating medication appropriateness in U.S. geriatric care and research. It is regularly updated, and exposure defined by Beers is associated with adverse outcomes in older adults, such as hospitalization and mortality [14,15]. For each participant, we identified any medication that met the Beers Criteria (applying disease-specific and renal recommendations when relevant data were available) and summarized outcomes as the proportion with more than one PIM out of all identified issues.

### 2.5. Outcomes

Depending on when the participants have their follow-up appointments with their healthcare providers, a clinical pharmacist contacted them regarding the recommendations made during the MTM events to inquire whether their providers had modified their therapeutic plans based on the recommendations or whether the participants themselves had made behavioral changes. For participants who did not have scheduled appointments during our follow-up calls, the team would reach out to them again approximately three to six months later to inquire about the outcomes of our recommendations. [However, oftentimes, they did not answer our calls.]

This study examined a list of quantitative variables: (1) process outcomes (e.g., the number of participants reached, the time spent in each session, and the types of interventions delivered during the community health events); (2) impact outcomes (e.g., captured through follow-up assessments, participants’ reports on the utilization of recommendations provided during the encounters, and primary care clinicians’ acceptance of our recommendations); and (3) participants’ satisfaction after receiving clinical pharmacy services (e.g., measured through post-encounter surveys where participants rated various aspects of their encounters). These three groups of outcomes were selected by the first author based on his experiences in program evaluation, consultation with his public health colleagues, and program evaluation concepts from this publication [16]. For this reason, the outcome measures were not tested. As a descriptive pilot, all outcomes are succinctly summarized using counts, percentages, and, where appropriate, means and standard deviations.

Two levels of impact outcomes designed for this study were: (1) Impact Rate—Participants Performing Self-Modification Based on the Recommendations, and (2) Impact Rate—Participants’ Primary Care Provider/Clinician Accepting Our Recommendations. These impact rates depended on the Successful Follow-Up Rate, which was determined by data collected in subsequent conversations with the participants.

### 2.6. Sample Size & Statistical Methods

This study utilized a convenience sampling method, relying on the residents’ willingness to consider our recommendations and their desire to complete the post-encounter survey. As this evaluation was descriptive, the outcomes were analyzed using descriptive statistics through Microsoft Excel, capturing process and impact outcomes.

During data collection, missing data primarily resulted from participants who did not complete the post-encounter questionnaire and could not be reached for follow-up calls regarding the implementation of recommendations provided during the MTM sessions. To address this, we utilized the following approaches:

- Missing responses were recorded as “no response available” and excluded from calculating specific outcome measures (such as participant satisfaction) that required participant feedback.
- For measures requiring a follow-up response (such as participant-reported behavioral changes or clinician acceptance of recommendations), percentages were calculated based on the available data rather than the initial number of participants enrolled.
- Given the voluntary nature of follow-up participation, we acknowledge that missing data can impact our overall study outcomes. To address this issue, future studies will implement structured follow-up strategies, such as scheduled calls or text reminders, to improve participant retention.

### 2.7. Bias

The following steps, summarized in Table 1, were considered to minimize potential sources of bias in this evaluation.

### 2.8. Participant Satisfaction Survey

Immediately following each MTM encounter, participants completed a concise satisfaction survey crafted by the team to gauge the acceptability and perceived utility of the service within this community context. Survey items used 3- or 5-point Likert scales with standardized anchors (See Supplemental Materials for details). To address the potential challenges of low health literacy among respondents, the staff opted to administer the survey in an oral format, ensuring that all responses were meticulously recorded verbatim. This approach was implemented to maintain anonymity and prevent any potential linking of responses to personal identifiers.

Because the instrument was designed for rapid use at events and satisfaction was a secondary outcome, no formal psychometric validation was undertaken. We treated the scales as ordinal and summarized responses descriptively. Consequently, findings should be interpreted as descriptive indicators of acceptability rather than validated scale outcomes. Immediate post-encounter administration may have introduced social desirability bias.

### 2.9. Follow-Up Calls

The follow-up calls from our team were scheduled after the participant’s next clinic visit with their clinicians or at ~3–6 months post-encounter if no visit had been planned. The follow-up calls were made by only one clinical pharmacist due to staffing shortages. The intended cadence was to have three call attempts on different days and times within a two-week window for each follow-up point. The team’s ability to consistently execute this final step was compromised by the staffing shortage. Since only one person made the follow-up calls, no standardized script was developed.

### 2.10. Use of Artificial Intelligence Tools

Generative AI tools, including ChatGPT 4o and 5, were employed to identify relevant references for this manuscript. No identifiable or participant-related information was uploaded. Furthermore, Grammarly was used to enhance the quality of the language. It is important to note that this tool did not generate any new scientific content, conclusions, or references. All rephrased text was meticulously verified and edited by the authors.

## 3. Results

This study employed process and impact evaluations to assess the implementation and immediate effects of the CHA. The process evaluation involved assessing activities and reach. The impact evaluation included (1) behavioral changes and (2) clinicians’ acceptance of our recommendations.

**Figure 1:**
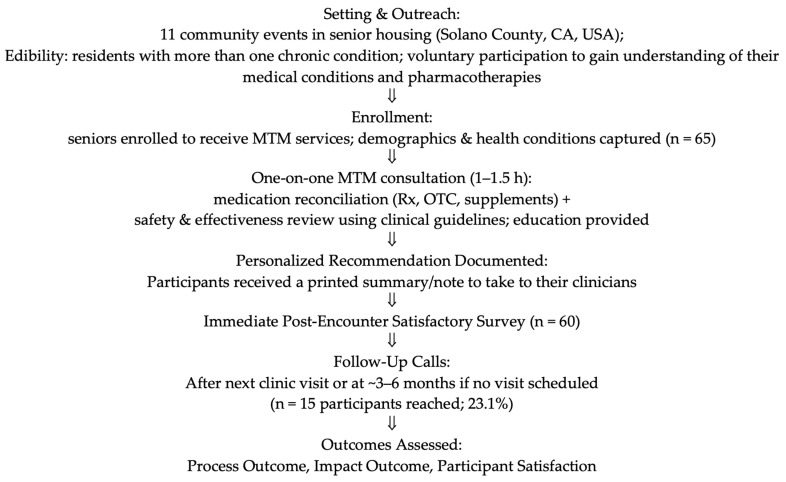
Protocol Schema and Participant Flow (January 2022 to August 2023).

From January 2022 to August 2023, we served 65 participants across ten community health events. Table 2 summarizes the baseline demographics of these participants, who had an average age of 74.5 years. Figure 1 summarizes the protocol schema and participant flow with outcomes.

The MTM sessions identified 118 medication-related issues (summarized in Table 3), including expired medications, untreated conditions, therapeutic duplications, medications without an indication, drug–drug interactions, contraindications, Beers Criteria (defined as “an explicit list of PIMs [Potentially Inappropriate Medications] that are typically best avoided by older adults in most circumstances or under specific situations, such as in certain diseases or conditions”) [13,17], and adverse drug reactions. Table 4 shows details on impact outcomes. One key result is that nearly a quarter of the evaluated items (combining prescription medications and OTC items) would require some level of intervention from the team.

**Figure 2:**
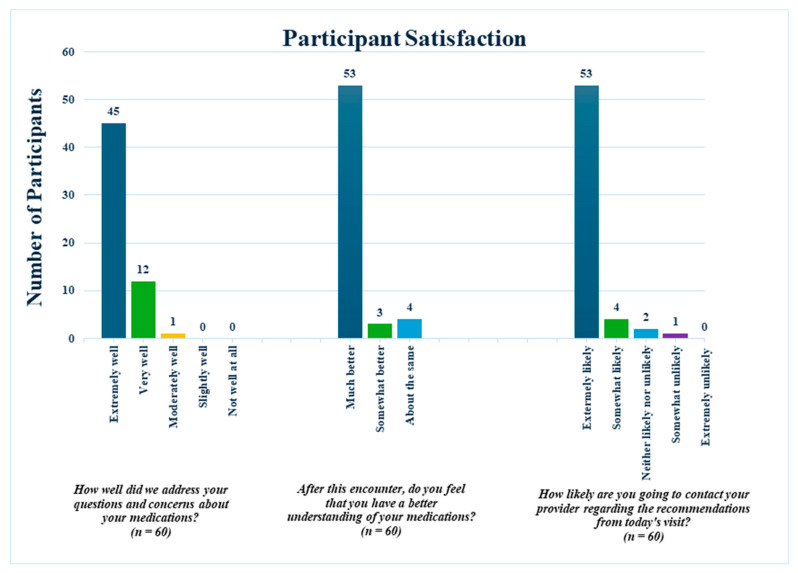
Participant Satisfaction.

Post-encounter surveys were conducted to gather participant satisfaction, with 60 participants completing the questionnaire. Figure 2 shows the results of participant satisfaction, which included very positive feedback. Of the 60 participants who completed the post-survey, 45 (75%) reported that their questions and concerns about their medications were addressed “extremely well,” and 12 (20%) reported “very well.” Fifty-three participants (88%) reported feeling “much better” regarding their understanding of medications. Lastly, 53 participants reported that contacting their clinicians regarding the recommendations from their Pharm2Home encounters was “extremely likely.”

Table 3 presents routine data collected from all community events, summarizing the key impacts. One of the impact rates was self-modified behaviors after the initial encounter at 27.1% ([the sum of the Number of Modifications Made by Participants + the sum of the Number of Recommendations Accepted by Participants’ Primary Care Providers (PCPs)/Clinicians at a Subsequent Medical Appointment] divided by the sum of the Number of Recommendations Made or Education Points Shared), and 31.2% of the recommendations brought in by the participants to their clinicians were accepted (the sum of the Number of Recommendations Accepted by Participants’ PCPs/Clinicians at a Subsequent Medical Appointment divided by the sum of the Number of Recommendations P2H Encouraged Participants Bringing to Their PCPs/Clinicians). A successful follow-up rate of 23.1% for collecting outcomes data limited these two impact outcomes. The effect on health outcomes has yet to be determined since we only had one intervention point. This study was designed as a proof-of-concept, initial implementation phase, so we did not collect any longitudinal data, which would require much more robust study support and funding.

## 4. Discussion

### 4.1. Statement of Key Findings

These preliminary results represent a significant step toward a multidisciplinary approach to community health, specifically focusing on older populations residing in low-income senior housing facilities. The CHA evaluated 491 medication and supplement items, identifying 118 interventions related to prescription medications and OTC/supplement items, which represents 24.0% of all items reviewed. Preliminary data show that MTM services and chronic disease education have been well received. Almost all participants are willing to elevate our recommendations to the next level, which could potentially impact their management of chronic diseases. The participants’ desire to communicate our recommendations to their clinicians for modifying their chronic disease management approaches indicates their trust in our clinical judgment and confidence in the encounters. MTM is a much-needed service for older adults, ensuring the continued safety and effectiveness of their pharmacotherapies, which is well-supported by literature [18].

With 31.2% of the recommendations accepted by participants’ clinicians—focusing on the safety and effectiveness of pharmacotherapies and supplements for chronic disease management—this indicates that the program not only underscores the importance of clinical pharmacist-led interventions but also highlights the potential for patient advocacy and empowerment in healthcare decision-making. Compared to a study specifically examining the use of high-risk medications and statins for primary prevention among Medicare Part D beneficiaries with diabetes, the overall acceptance rate of prescribers for pharmacists’ recommendations was 35% [19]. Considering our team’s role at community events was to train senior participants on advocating for themselves regarding their pharmacotherapies and overall chronic disease management, an acceptance rate approaching one-third was an encouraging and promising initial outcome. Pharmacist-led interventions in older adults, beyond mere acceptance, have been linked to enhanced medication adherence and a decrease in medication-related adverse effects: meta-analyses show improvements in adherence and reductions in adverse drug reactions [20,21,22].

The findings from this evaluation effort align with existing literature, indicating the positive impact of clinical pharmacist-led community interventions for chronic disease management [23]. These interventions aim to advance health equity among older persons [24].

### 4.2. Strengths: Reflecting on Aligning with Health Equity

Our initiative aligns with the goal of health equity by addressing key barriers to healthcare access and empowering underserved populations. The CHA is designed to provide targeted support to seniors living in affordable housing communities who encounter systemic inequities in healthcare access and utilization. Furthermore, our emphasis on participant education aligns with research demonstrating that structured teach-back enhances comprehension and safety for individuals with limited health literacy, which is particularly pertinent in a senior housing population [25,26].

Our program aims to eliminate logistical barriers, such as transportation challenges, by placing clinical pharmacists directly within senior housing facilities. This community-based approach ensures that seniors can access high-quality MTM regardless of socioeconomic status. Our work prioritizes improving health literacy and self-advocacy among seniors who struggle to understand their medications and conditions. By tailoring education to the needs of this population, Pharm2Home equips participants with the tools and knowledge necessary to engage effectively with their healthcare providers and make informed decisions. This approach empowers participants and directly supports health equity by addressing disparities in health knowledge and self-management capabilities.

### 4.3. Weaknesses

While the results are promising, this study is limited to an older adult cohort, underscoring the need for a broader scope that encompasses diverse age groups to advance health equity further. Although the current outcomes provide minimal insight into the potential long-term effects on health outcomes, this data offers valuable insights into the future of community health efforts, particularly focusing on the senior population. Programs like Pharm2Home hold significant potential for shaping future healthcare practices in the community beyond traditional medical settings [27]. This suggests that community outreach programs incorporating integrated multidisciplinary health teams can enhance the effectiveness of pharmacotherapy and improve patients’ health-related knowledge. Future efforts should focus on scaling up and making the interventions more applicable across diverse demographic segments. The limitations of external validity and the lack of insight into subsequent clinical outcomes arise from the inclusion of only older adults, leaving us unclear about how this intervention may affect younger adults and youth.

Additionally, we are uncertain about the degree of interventions needed to induce meaningful clinical outcomes. Future studies and evaluations may clarify whether the impact of a single encounter within a community setting versus multiple visits generates similar results and whether this model can be applied to other health conditions. Further evaluations with larger samples, including younger adults, and an extended follow-up period are warranted to determine the impact of such community health efforts.

While the Pharm2Home Initiative demonstrated promising outcomes in enhancing health education and medication management among participating seniors, our approach encountered design limitations, including its non-randomized structure, which increased the risk of selection bias. Furthermore, we conducted minimal follow-up efforts due to staff shortages, which may have potentially affected the quality of the documented outcomes.

### 4.4. Interpretation: Implications for Practice

The CHA findings offer several actionable insights on enhancing the roles of clinical pharmacists in chronic disease management within community settings.

(1) Our results underscore the importance of involving pharmacists in direct patient education and care management, extending beyond their traditional roles in pharmacies. Health promotion programs should integrate clinical pharmacists into community centers, senior homes, and local clinics, where they can provide personalized consultations and empower patients through education.
(2) Given our participants’ high appreciation for personalized education sessions, health systems should consider developing targeted educational materials that address the specific needs and common conditions of the senior population. These programs should focus on managing medication, understanding prevalent chronic conditions, and navigating the healthcare system effectively.
(3) Encouraging self-management is a key component of the P2H Initiative. Future programs should incorporate tools and resources that help seniors track their medication schedules, understand their prescriptions, and identify when to seek medical advice. This would promote greater independence and confidence in managing their health and wellness.

These adaptations may help achieve better health outcomes and align with broader public health goals of increasing accessibility, reducing healthcare costs, and enhancing the quality of life for seniors.

### 4.5. Potential Pathways to Improve Communication and PCP Acceptance of Recommendations

Our pilot mainly depended on participant-mediated communication (a printed summary taken to clinic visits), which probably limited downstream adoption. Specifically, 23.1% of participants were successfully contacted for follow-up, and among the recommendations that participants reported sharing with their clinicians, 31.2% were accepted. These figures highlight the need for more effective, closed-loop P2H Team-PCP communication to improve the acceptance of pharmacotherapeutic recommendations.

Participant-Level Strategies. The primary objective is to equip the messenger (the participant) with the necessary information to emphasize the most significant changes during their visit with their PCP. For instance, they should include a wallet-sized medication card that prominently displays high-risk items (such as the Beers Criteria) and the single most crucial medication change to request. This approach aims to minimize message dilution during brief visits. Furthermore, incorporating a simple checklist (“Request X medication. Request to recheck Y lab.”) can facilitate concrete requests.

Clinician-Facing Strategies. The primary objective is to facilitate the acceptance of the request. For instance, we can revise our current standardized post-encounter summary to a more practitioner-friendly format:

- Situation: A concise summary of the patient’s problem.
- Background: Information regarding relevant medications and contraindications.
- Assessment: Concerns regarding safety and effectiveness, Beers violations, and any drug–drug interactions.
- Recommendations: Specific actions, monitoring, and follow-up intervals.
- This revised summary includes clear guideline citations and, where applicable, renal dose or drug–drug interactions to reduce cognitive load. For high-risk items (e.g., Category D interactions, clear Beers violations), schedule a same-week P2H-to-PCP call to discuss the most critical change and agree on a monitoring plan.

Together, layering these two strategies could enhance communication accuracy and boost clinician acceptance beyond the initial rate seen so far.

### 4.6. Looking into the Future: Implications for Future Research

Despite the limitations, the findings suggest that clinical pharmacist-led interventions in community settings can help seniors enhance their approaches to health maintenance. There is a need for longitudinal studies to assess the long-term impacts of clinical pharmacist-led interventions on patient health outcomes. Such research should aim to quantify improvements in health literacy, medication adherence, and clinical outcomes over extended periods. Additionally, understanding the long-term health outcomes remains imperative to validate the potentially sustained impacts of community-integrated health programs, such as the Pharm2Home Initiative. These results provide a foundation for community health efforts centered on seniors, utilizing an integrated healthcare team to enhance health literacy and optimize pharmacotherapies.

Although this study is preliminary, our effort supports the hypothesis that community-based interventions can empower participants to improve their overall health outcomes related to chronic disease management. Additional studies are needed to confirm this hypothesis further and determine how such efforts may be applied to managing other chronic conditions across different age groups.

Future investigations are crucial to determine whether the benefits of this community-based approach extend to improving clinical outcomes, self-management, and self-advocacy in diverse chronic conditions, particularly among clinically complex older individuals. Future endeavors should prospectively monitor objective indicators (e.g., A1c, blood pressure, falls, emergency department visits) and de-prescribing endpoints. Recent systematic reviews and meta-analyses suggest that medication review and de-prescribing can reduce drug burden and potentially impact downstream outcomes [28,29,30].

## 5. Conclusions

Although the Pharm2Home Initiative Community Health Arm has yielded some promising preliminary results in enhancing participants’ understanding of their chronic conditions and improving medication management, further research is necessary to assess the long-term impact of the intervention on health outcomes and the management of chronic diseases.

Our platform involved clinical pharmacists collaborating with senior housing facilities to address medication-related concerns and empower seniors to advocate for their health needs with their healthcare providers. Although the findings are considered exploratory and the impact remains to be determined in longer-term, larger studies, this evaluation approach has highlighted the practicality and efficacy of community health interventions led by clinical pharmacists in enhancing medication comprehension and chronic disease self-management among vulnerable older adults.

## Figures and Tables

**Table 1:** Summary of Potential Biases.

Selection Bias	Participating in the MTM sessions was voluntary, which could potentially introduce self-selection bias. To mitigate this concern, all eligible seniors residing in the housing facilities were invited to participate through standardized outreach efforts, thereby ensuring a board and representative sample.
Recall Bias	Recall bias, which occurs when participants share information based on their memory rather than objective facts, could have influenced their responses during and after the encounters. To address this, the team implemented a check-and-balance system by incorporating subjective and objective information during the encounters. However, during the encounters, the team was limited to responding to what the participants were willing to share, which posed a challenge in determining whether recall bias was present.
Confounding Bias	Given the influence of various factors beyond our team’s interventions on chronic disease management, potential confounders like healthcare access, baseline health literacy, and socioeconomic status were present. Whenever possible, our findings were contextualized within the broader healthcare context. Future studies may include control groups or more structured comparisons to validate our results.
Measurement Bias	The use of study-specific forms and short surveys without validation may have introduced measurement bias.

**Table 2:** Participant Demographics.

n = 65			
**Mean Age (SD)**	74.5 (7.6) years old		
**Gender**		**Educational Level**	
Male	19 (29.2%)	8th Grade or Less	10 (15.4%)
Female	46 (70.8%)	Some High School	8 (12.3%)
Nonbinary/Transgender/Bisexual/Prefer not to say	0 (0%)	High School Degree	18 (27.7%)
**Race**		Some College	22 (33.8%)
Asian	19 (29.2%)	Bachelor’s Degree	6 (9.2%)
African American	19 (29.2%)	Prefer Not To Say	1 (1.5%)
Caucasian/White	16 (24.6%)	**Marital Status**	
Native Hawaiian	1 (1.5%)	Married	13 (20%)
Other	9 (13.8%)	Single	20 (30.1%)
**Ethnicity**		Divorced/Separated	16 (24.6%)
Hispanic/Latino	10 (15.4%)	Widowed	15 (23.1%)
Non-Hispanic/Latino	55 (84.6%)	Prefer Not To Say	1 (1.5%)
**Primary Language**		**Hospitalization/Surgery < 12 Months**	
English	40 (61.5%)	Yes	28 (43.1%)
Tagalog	11 (16.9%)	No	37 (56.9%)
Spanish	6 (9.2%)	**Immunized ^a^**	
Mandarin	2 (3.1%)	Yes	13 (20%)
Punjabi	2 (3.1%)	No	51 (78.5%)
Other	4 (6.2%)	Unsure	1 (1.5%)
**Insurance**		**COVID-19 Vaccines (Initial Series)**	
Medicare	20 (30.8%)	Yes	56 (86.2%)
Medicaid	6 (9.2%)	No	9 (13.8%)
Both Medicare and Medicaid	34 (52.3%)	**COVID-19 Booster Vaccine**	
Commercial	5 (7.7%)	Yes	51 (78.5%)
		No	14 (21.5%)

^a^ Immunized with the following vaccinations: influenza, pneumonia, MMR (Measles, Mumps and Rubella), Tdap (tetanus, diphtheria, and pertussis), COVID-19 (+booster, updated bi-valent booster), Shingles, Hepatitis A, and Hepatitis B.

**Table 3:** Process Outcomes.

**Process Outcomes (Interventions Made During the Encounters)**	
# of Over-The-Counter (OTC) and Supplement Items Evaluated	106
# of Prescriptions Evaluated	385
Total Number of Items Evaluated	491
**Issues Identified (Total = 118)**	
# of Expired Medications	26 (5.3% **^a^**)
# of Untreated Conditions	21 (4.3% **^a^**)
# of Therapeutic Duplications	8 (1.6% **^a^**)
# of Medications without an Indication	20 (4.1% **^a^**)
# of Category D ^b^ Drug–Drug Interactions	16 (3.3% **^a^**)
# of Contraindications	4 (0.81% **^a^**)
# of Medications on Beers Criteria	14 (2.8% **^a^**)
# of Adverse Drug Reactions Identified	9 (1.8% **^a^**)
	118/491 = 24.0%
**Consultations (Total = 126)**	
Medication Counseling	61 (48.4%)
Immunization Counseling	23 (18.3%)
Lifestyle Counseling	21 (16.7%)
Other Counseling ^c^	21 (16.7%)

# = number; **^a^** = with 491 items reviewed as the denominator; ^b^ Category D Drug–Drug Interactions = when two drugs interact with each other, either increasing or decreasing the one drug’s concentration, which requires either dose adjustment or agent modification (followed by frequent monitoring to minimize and avoid toxicity) [13]; ^c^ i.e., healthcare system navigation, mental health, lab interpretations, etc.

**Table 4:** Impact Outcomes.

**Impact Outcomes (Data Collected Post-Encounter)**						
**Event**	**Number of Participants Served**	**Number of Recommendations Made** **or** **Education Points Shared**	**Number of Participants Followed-Up**	**Number of Modifications Made by Participants**	**Number of Recommendations P2H Encouraged Participants Bringing to Their PCPs/Clinicians**	**Number of Recommendations Accepted by Participants’ PCPs at a Subsequent Medical Appt**
1	12	34	8	10	23	16
2	7	35	2	8	14	6
3	8	26	1	0	15	2
4	7	19	0	0	9	0
5	3	0	0	0	0	0
6	2	11	2	1	9	5
7	6	19	0	0	17	0
8	3	10	0	0	4	0
9	6	15	2	4	2	0
10	4	7	0	0	0	0
11	7	16	0	0	0	0
Sum	65	192	15	23	93	29

Summary of Impacts: Successful Follow-Up Rate (A Subsequent Conversation with the Participant): 15/65 (23.1%). Impact Rate—Participants Performing Self-Modification Based on Recommendations: 23/192 (12.0%). Impact Rate—Participant’s Primary Care Provider/Clinician Accepting Recommendations: 29/93 (31.2%).

## Data Availability

The data presented in this study are available on request from the corresponding author due to privacy concerns.

## Supplementary Materials

The following supporting information can be downloaded at: https://www.mdpi.com/article/10.3390/pharmacy13050141/s1, File S1: Intake Form, Visit Summary, and Satisfactory Survey.
