# Supplementary material for: Community Health Empowerment Through Clinical Pharmacy: A Single-Arm, Post-Intervention-Only Pilot Implementation Evaluation
Source: Pharmacy (Basel). 2025 Oct 1;13(5):141. doi: 10.3390/pharmacy13050141 (PMC12567540; doi:10.3390/pharmacy13050141)
Supplement: Supplementary file 1 [file pharmacy-13-00141-s001.zip › pharmacy-3798375-supplementary.pdf]

pharmacy

MDPI

Supplemental Materials File S1: Intake Form, Visit Summary, and Satisfactory Survey.

(1) Intake Form

- Event Site Name
- Enter Today's Date (e.g., 01/01/2023)
- START TIME (*use military time - 09:15 or 13:15, etc.*)
- Please enter the **names of the students** who are conducting the MTM intervention.
- Participant's Name
- Please choose which language is used to conduct the MTM:
    - English
    - Spanish
    - Other
- READ TO THE PATIENT (speak clearly): We are here to evaluate all your medications, help you understand your medications, and answer any questions you may have about them. During this encounter, we will ask you some questions. The information we collect about you, including your health, interventions, and any information that could be traced back to you, will not be collected. If you do not want to answer a question or wish to end the encounter at any time, please let us know, and we will accommodate your request. Are you willing to participate?
    - Yes (signature obtained)
    - No (please fill out why)
- Collect from the patient, as they may have had it done at the MOBEC station, or kindly ask if they would like to have their BP checked.
    - SEATED BP (mmHg)
    - REPEATED SEATED BP (mmHg) if >130/90
    - Heart rate (bpm)
    - REPEATED heart rate (bpm)
    - STANDING BP (mmHg) [if patient unable to stand up or requires support, then skip and enter N/A]
    - REPEATED STANDING BP (mmHg)
- Collect the information from the patient, as they may have already had it done at the MOBEC station. If none, please leave blank.
    - Blood Glucose (mg/dL)
    - Hemoglobin A1c (%)
    - CHECK THIS BOX IF patient is FASTING (no food, snack, drinks other than water in last 8 hours)
- Which of the following genders do you identify as?
    - Male
    - Female
    - Non-binary/third gender
    - Transgender
    - Bisexual
    - Prefer not to say
- What is your age? (Please only enter a number)
- Which of the following best describes your race?
    - American Indian/Alaska Native
    - Asian
    - African American/Black
    - Caucasian/White
    - Native Hawaiian or Pacific Islander
    - Prefer not to say
    - Other
- Are you of Hispanic or Latino ethnicity?
    - Yes
    - No
    - Prefer not to say
- What is your primary language?
    - English
    - Spanish

    - Tagalog
    - Cantonese
    - Mandarin
    - Vietnamese
    - Hindi
    - Punjabi
    - Other
- What insurance do you currently have?
    - Medicare
    - Medi-Cal
    - Commercial
    - Uninsured
    - Tricare
    - Medicare & Medi-Cal
    - Other
- What is your marital status?
    - Married
    - Single
    - Widowed
    - Divorced/Separated
    - Prefer not to say
- What is your highest level of education?
    - 8th grade or less
    - Some high school (till 12th grade)
    - High school graduate/GED
    - Some college or an associate's degree
    - Bachelor's degree or higher
    - Prefer not to say
    - Other
- Do you smoke, vape, or use recreational drugs? (Enter number of cigarettes per day if applicable)
    - Yes
    - Sometimes
    - Never smoker
- If they smoke or used to smoke, select the following (if not, skip question):
    - Current
    - Former
    - Vape
    - Recreational Drugs
    - Prefer not to answer
    - Never
- Do you currently drink alcohol? If so, please specify the number of drinks per day or per week.
    - Yes
    - No
- Do you drink caffeinated beverages? If so, please list the number of drinks per day and their type, if applicable.
    - Yes
    - No
- Are you currently pregnant?
    - Yes
    - No
    - Not applicable
- What allergies (i.e., medications, food, etc) do you have? If you have an allergy to ____, what reaction did you experience?
    - Medications
    - Food

  - Latex
  - Animals/Insects
  - Environmental (i.e., dust, plants, etc.
  - NONE
- Have you been recently hospitalized, visited the ER, or had a recent surgery in the last 12 months? *If yes to any of the above, please enter the reason in the text box.*
  - Yes
  - No
- Which of the following chronic conditions did your doctor and/or health care professional tell you that you have?
  - Endocrine Disorders
    - Atypical diabetes (i.e., LADA, MODY, secondary diabetes)
    - Type 1 Diabetes
    - Type 2 diabetes
    - Hyperthyroidism
    - Hypothyroidism
    - Other
  - Cardiovascular Disease
    - Atrial Fibrillation
    - Arrhythmias
    - Dyslipidemia
    - Hyperlipidemia
    - Heart Failure
    - Hypertension
    - Myocardial Infarction
    - Stroke
    - Pulmonary Embolism
    - Deep Vein Thromboembolism
    - Other
  - Respiratory Disorder
    - Asthma
    - COPD
    - Pulmonary Hypertension
    - Other
  - Neurological/Psychological Disorder
    - Alzheimer's Disease
    - Anxiety
    - Bipolar Disorder
    - Depression
    - Dementia
    - Insomnia
    - Schizophrenia
    - Attention-deficit/Hyperactivity Disorder
    - Parkinson's Disease
    - Epilepsy
    - Multiple Sclerosis
    - Headaches and Migraines
    - Restless Leg Syndrome
    - Other
  - Chronic Kidney Disease
    - End-stage renal disease
    - On Dialysis
    - Other
  - Bone, Joint, Gout Disorders
    - Osteoporosis
    - Osteoarthritis

        - Rheumatoid Arthritis
        - Gout
        - Other
    - Reproductive Disorders
        - Benign Prostatic Hyperplasia (BPH)
        - Erectile Dysfunction
        - Menopause
        - Other
    - Gastrointestinal Disorders
        - Incontinence
        - Constipation
        - Diarrhea
        - GERD / Heartburn
        - Inflammatory Bowel Disease
        - Other
    - Infectious Diseases
        - Hepatitis B
        - Hepatitis C
        - HIV
        - Other
    - Miscellaneous Conditions
        - Anemia
        - Hearing Difficulty
        - Cancer
        - Glaucoma
        - Cataract
        - Obesity
        - Chronic Pain (specify location)
        - Other
- What vaccines have you received as of today?
    - Influenza
    - Pneumococcal (PCV13)
    - Pneumococcal (Prevnar 20)
    - Pneumococcal (PPSV23)
    - MMR
    - TDaP
    - Shingles (Shingrix) 2-dose series
    - Zostavax (old Shingles vaccine - 1 dose)
    - Varicella
    - Hepatitis A
    - Hepatitis B
    - COVID-19 Primary Series (enter which one and when if they remember)
    - COVID-19 Primary Series (enter which one and when if they remember)
    - Other Vaccines
    - Not sure
- What concerns (if any) do you have about your health conditions?
- How can we assist you in addressing your primary concerns about your health conditions? (Patient Goals)
- Do you have a good understanding of your medications? [*PARTICIPANT'S PERSPECTIVE]*
    - Yes
    - No
    - Somewhat
- Do you know what your medications are for? (This is where you ask them to tell you (*HCP perspective*); ***If there are medications that the patient states they do not know, please list which ones and educate).***

(2) Visit Summary (Sample Clinical Note to Be Taken to Clinicians)

Sample Note

Thank you for attending the Pharm2Home Medication Therapy Management Event on [DATE]. If you have any questions or concerns, please get in touch with our Pharm2Home Team at [Phone Number].

Below, we have some recommendations based on our review today. Please bring this to your next medical visit and discuss the recommendations.

Primary Care Provider: [Name of the Physician]
Insurance: Medicare/Medi-Cal

Blood Pressure readings at today's event:
BP right arm 114/55; Pulse 70
Recheck after 15 mins BP right arm 105/60; Pulse 70
Per caregiver, home BP is usually around 136/84
Allergies: codeine (foot flares/swelling)
Hospitalizations/Surgery: right knee replacement; had DVT

82-year-old female with PMH of type 2 diabetes, hypertension, neuropathy, history of pulmonary embolism, osteoporosis, osteoarthritis in the shoulder, and history of knee replacement. She is here for a medication review. Discussed all medications and listed below with indications. The participant is having labs completed today and has been advised to follow up on potassium, electrolytes, and kidney function (eGFR, UACR) to assess whether her medications are dosed appropriately.

Her DBP is slightly below 60, and the recheck was back to 60. Will need regular BP monitoring at home to ensure that BP is not below 90/60, as that is considered low blood pressure. She drinks about 1 to 2 cups of coffee, Pure Leaf black tea, usually more often during the summer. Blood sugar levels range from 120 to 140 and are generally below 150, according to the caregiver. She's been attending Success with Diabetes (6-week course) to learn more about diabetes management, nutrition, and medications.

Diet:
Breakfast: scrambled eggs, frozen waffles, no sugar syrup; sometimes Jimmy Dean sausages that cause diarrhea, but coffee helps constipation
Lunch: banana
Dinner: meats, vegetables, mac & cheese
Snacks: fresh snap peas, sometimes oranges, kiwi, but worried about sugar content

Plan/Educational Points:

1. Check your blood pressure at least once a day (preferably 2 hours after taking your medications).
2. Follow up on your potassium labs and kidney function as you are taking supplementation.
3. Recommend checking blood sugar levels in the morning before meals and 2 hours after the largest meals. General targets are fasting between 80 and 130, and random or 2 hours after a meal is less than 180. Your doctor may set additional targets if deemed necessary.

Medications confirmed with bottles:

1. Pradaxa 150mg 1 capsule by mouth 2 times a day for thinning blood to prevent clots [PCP]
2. Gabapentin 300mg 1 capsule by mouth in the morning and one capsule at bedtime for nerve pain [PCP]
    a. **Used to be 300mg AM 600 mg at night
3. Memantine 5mg 1 tablet by mouth 2 times a day for memory [PCP]
4. Losartan 50mg 1 tablet by mouth daily [PCP]
5. Potassium chloride ER 8mEq one tablet by mouth daily with a meal, always take with furosemide (Lasix)
6. Furosemide 20mg 1 tablet by mouth every morning (discontinue triamterene/hydrochlorothiazide 37.5mg/25mg) [PCP]
7. Glipizide 5mg 1 tablet by mouth 2 times a day, 30 mins before breakfast and dinner for blood sugar/diabetes management (replaces glimepiride)

Over-the-Counter:

Lidocaine patches, one patch for 12 hours as needed for low back pain
Vitamin B12 500mcg one tablet by mouth a day

Has bottles but NOT TAKING at the moment as it was stopped by the provider:

- Gabapentin 600mg 1 tablet by mouth 2 times a day

Next Appointment: The participant will receive lab results today, and then a follow-up will be scheduled with the PCP.

(3) Satisfactory Survey

TOURO UNIVERSITY
CALIFORNIA

How well did we address your questions and concerns about your medications?

- ○ Not well at all
- ○ Slightly well
- ○ Moderately well
- ○ Very well
- ○ Extremely well

After this encounter, do you feel that you have a better understanding of your medications?

- ○ Much better
- ○ About the same
- ○ Somewhat better

How likely are you going to contact your provider regarding the recommendations from today's visit?

- ○ Extremely unlikely
- ○ Somewhat unlikely
- ○ Neither likely nor unlikely
- ○ Somewhat likely
- ○ Extremely likely

We would like to receive your feedback about today's interventions and encounter. Would you recommend this service to your family and friends? Why or why not? (please enter patient feedback)

- ○ Yes
- ○ No
